# Associations of premorbid adjustment with type and timing of childhood trauma in first-episode schizophrenia spectrum disorders

**DOI:** 10.4102/sajpsychiatry.v27i0.1639

**Published:** 2021-06-22

**Authors:** Anna M. Smit, Sanja Kilian, Robin A. Emsley, Hilmar K. Luckhoff, Leslie Swartz, Soraya Seedat, Laila Asmal

**Affiliations:** 1 Department of Psychiatry, Faculty of Medicine and Health Sciences, Stellenbosch University, Cape Town, South Africa; 2 Department of Psychology, Faculty of Arts and Social Sciences, Stellenbosch University, Cape Town, South Africa

**Keywords:** childhood trauma, premorbid adjustment, first episode schizophrenia spectrum disorders, academic and social functioning, early adolescence

## Abstract

**Background:**

Childhood trauma may contribute to poorer premorbid social and academic adjustment which may be a risk factor for schizophrenia.

**Aim:**

We explored the relationship between premorbid adjustment and childhood trauma, timing of childhood trauma’s moderating role as well as the association of clinical and treatment-related confounders with premorbid adjustment.

**Setting:**

We conducted a secondary analysis in 111 patients with first-episode schizophrenia (FES) disorders that formed part of two parent studies, EONKCS study (*n* =73) and the Shared Roots study (*n* =38).

**Methods:**

Type of childhood trauma was assessed with the Childhood Trauma Questionnaire, short-form and premorbid adjustment using the Premorbid Adjustment Scale. Timing of childhood trauma was assessed using the Life Events Checklist and life events timeline. Linear regression analyses were used to assess the moderating effect of timing of childhood trauma. Clinical and treatment-related confounders were entered into sequential hierarchical regression models to identify independent predictors of premorbid adjustment across key life stages.

**Results:**

Childhood physical neglect was associated with poorer premorbid academic functioning during childhood and early adolescence, and poorer premorbid social functioning during early and late adolescence. By hierarchical regression modelling (*r*^2^ = 0.13), higher physical neglect subscale scores (*p* = 0.011) independently predicted poorer premorbid social adjustment during early adolescence. Timing of childhood trauma did not moderate the relationship between childhood trauma and premorbid functioning.

**Conclusion:**

In patients with FES, childhood physical neglect may contribute to poorer premorbid social functioning during early adolescence. This may provide us with an opportunity to identify and treat at-risk individuals earlier.

## Introduction

Childhood trauma exposure is associated with increased risk for the development of severe mental illnesses, including schizophrenia.^1,2^ Indeed, approximately 45% of individuals diagnosed with a psychiatric disorder report a history of childhood trauma as opposed to 33% of the population without a psychiatric disorder.^3,4^ Poor premorbid adjustment is frequently a precursor to schizophrenia and may reflect compromised neurodevelopment.^5,6^ Early life adversity, which may be associated with childhood trauma, has been linked to poorer premorbid adjustment in patients with schizophrenia.^7^

An association between childhood trauma and poorer premorbid adjustment across key developmental stages has also been replicated in some^5,8^ but not all^9,10^ studies. Stain et al.^8^ reported significant correlations between all childhood trauma types and premorbid social functioning in childhood and early and late adolescence in a first-episode psychosis sample. However, Trauelsen et al.^9^ failed to demonstrate an association between childhood trauma and any of the premorbid developmental periods in first-episode psychotic disorders. Chan et al.^10^ investigated a chronic schizophrenia sample and reported no associations between childhood trauma and premorbid social and academic functioning during any of the developmental stages.

A potentially important determinant of the effects of trauma exposure is the timing thereof. As far as we know, only one study to date has investigated the relationship between the timing of trauma exposure and premorbid adjustment and found that the timing of trauma exposure moderated its relationship with premorbid global functioning in schizophrenia. Those exposed to sexual and physical abuse (SPA) before the age of 11 suffered global premorbid functional impairment at all time points of the study, compared with an improvement of global functioning overtime for those exposed to SPA at a later age.^11^ Global functioning refers to relational, social, academic and occupational functioning.^12^ Furthermore, the influence of specific childhood trauma subtypes on specific domains of premorbid functioning in first-episode psychosis samples remains incompletely described.^8^ This may be particularly important in lower- to middle-income countries (LMICs) such as South Africa, where many children suffer from malnourishment, have poor parental supervision, live in overcrowded households, have no access to running water and fear for their safety because of high crime levels.^13^

In a recent study conducted on 77 patients with first-episode schizophrenia (FES), an association was found between physical neglect and poorer premorbid social functioning in early adolescence.^5^ However, that study did not consider the timing of early life trauma and included only one measure for childhood trauma, the Childhood Trauma Questionnaire, short-form (CTQ-SF). Two prior South African studies found that the physical neglect subscale of the CTQ-SF was not homogeneous and did not display stable factor loading and it was recommended to include more than one measure for childhood trauma.^14,15^ The physical neglect subscale was also found to load on different factors and may vary across cultures.^16,17,18^ One reason could be that physical and emotional neglect subscales are conceptually intertwined, making it difficult to differentiate between the two subscales.^16,19^

A number of clinical and treatment-related factors may impact the relationship between childhood trauma and premorbid functioning across multiple developmental periods A higher number of completed years of education in people with schizophrenia may be associated with less cognitive impairment^20^ and better premorbid academic and social functioning,^21^ whilst a longer duration of untreated psychosis (DUP)^22^ and earlier onset of illness have been found to be independent predictors of poorer social functioning in people with schizophrenia.^23^ On the one hand, there is also evidence that greater symptom severity is associated with poorer premorbid social and academic functioning.^24^ On the other hand, it has been reported that people with schizophrenia with a history of social substance use have better premorbid social functioning potentially because of less negative symptoms.^25^

The present study was conducted in a larger sample of patients (*n* = 111) with FES, as opposed to a previous study that included a smaller sample set (*n* = 77). The operational definition of FES was treatment naïve, first-episode psychotic patients, diagnosed with schizophrenia, schizophreniform disorder or schizo-affective disorder. We aimed to further elucidate the relationship between childhood trauma exposure and premorbid adjustment by interrogating the effects of trauma type and timing on both premorbid academic and social functioning across different developmental stages. It could be that childhood trauma impairs premorbid academic and social functioning, which in turn renders individuals with a genetic predisposition towards schizophrenia more vulnerable and a greater likelihood to develop schizophrenia. A better understanding of the specificity of early trauma can inform preventative measures. We hypothesised that (1) childhood trauma would be associated with poorer premorbid adjustment, (2) different trauma types would have differential relationships with premorbid adjustment, (3) timing of childhood trauma would significantly moderate the relationship between premorbid functioning and childhood trauma, with earlier exposure associated with worse premorbid adjustment across multiple developmental periods, (4) clinical and treatment-related factors (symptoms severity, DUP, education, substance use and age at study entry) would be significant confounders in the relationship between childhood trauma and premorbid adjustment.

## Materials and methods

### Study setting and selection of participants

This study forms part of two parent studies, the EONKCS study (*n* = 126) and Shared Roots study (*n* = 38). For this secondary data analysis we used 111 participants: 73 participants from the EONKCS study and 38 participants from the Shared Roots study. The parent studies were both prospective longitudinal non-comparative studies where we followed up people with FES who were treated with the lowest effective dose of flupenthixol decanoate medication over 12–24 months.^26,27,28,29,30,31^

Participants in the parent studies were recruited from community health clinics and from first admissions to Tygerberg and Stikland Hospitals in the broader Cape Town area. The inclusion criteria for both parent studies were as follows: men and women; aged 16–45 years; inpatients or outpatients; presenting with a first psychotic episode, confirmed by the Diagnostic and Statistical Manual of Mental Diseases, fourth edition, text revisions (DSM-IV TR) criteria for schizophrenia, schizophreniform or schizoaffective disorder. The exclusion criteria were serious general medical conditions that would have affected participation in the study: lifetime exposure to antipsychotic treatment for more than 4 weeks, educational level less than Grade 7 and current substance abuse/dependence. The inclusion criteria for the present secondary data analysis were all the participants from the parent studies who had data available on both premorbid adjustment and childhood trauma.

### Patient assessment measures

All patients in the parent studies were assessed using the Structured Clinical Interview for DSM-IV (SCID).^32^ In addition, study questionnaires were used to capture relevant socio-demographic data, including age, sex, gender, DUP, substance use, baseline illness severity and years of education. Information on DUP, substance use and years of education were obtained through collateral from family and caregivers and documented in clinical source notes. Urine drug screen results were used to verify information on substance use. Illness severity at baseline was obtained from the Positive and Negative Syndrome Scale (PANSS) baseline scores.

#### Childhood trauma

Childhood trauma exposure was assessed using the CTQ-SF.^33^ The CTQ-SF is consisted of 28 Likert-type questions and provides three subscale scores for abuse (sexual, physical and emotional abuse) and two subscale scores for neglect (physical and emotional neglect).^34^ Scores on these subscales range from 5 to 25 and the total scale score (sum of all five subscales) from 25 to 125.^35^ Good test–retest reliability was reported on the extended 70-item CTQ (Intra-class correlation coefficient [ICC]) = 0.88).^36^ However, Spies et al.^14^ investigated the psychometric properties of the CTQ-SF and advised that the physical neglect subscale should be used in conjunction with supplementary assessment tools because of poor internal consistency and homogeneity.

The minimisation/denial score of the CTQ-SF was not taken into account because patients with mental illnesses tend to report more frequently on past traumatic events.^37^ We further assessed the type and timing of traumatic events with the Life Events Checklist (LEC-5), a self-report measure designed to screen for potentially traumatic events in a respondent’s lifetime.^38^ The LEC-5 was utilised to verify information on the childhood trauma subscales and in particular the physical neglect subscale of the CTQ-SF. We captured information on the timing of life events across the participant’s lifespan from a life events timeline because the CTQ-SF does not capture information on the specific timing of events.

#### Premorbid adjustment

The Premorbid Adjustment Scale (PAS) was administered by a research assistant or investigator on the patients and supplemented with collateral information from primary caregivers. The PAS has inter-rater reliability with an estimated range of 0.74–0.85.^39^ The PAS is used to measure functioning about peer relationships, sociality, adaption to school, school performance and socio-sexual aspects across four developmental periods: childhood (up to 11 years of age), early adolescence (12–15 years of age), late adolescence (16–18 years of age) and early adulthood (older than 19 years of age). There is also a general section that gathers information on changes in work and school performance, the highest level of education, and quality of life that was not included in the present analysis. Premorbid Adjustment Scale items are scored from 0 to 6, and higher item scores are indicative of poorer premorbid adjustment. The items per subscale are summed and divided by the sum of the highest possible score for items completed. The average of the subscales scores for all subscales comprises the total scale score. As academic and social domains are distinct components,^40^ these domains were studied separately across the developmental life stages of interest. As previously described by Allen et al.,^41^ we omitted data from the adulthood developmental period as this may have been affected by early manifestations of the illness.

### Ethical considerations

Ethical approval for the two parent studies and the present secondary data analysis were obtained from the Health Research Ethics Committee of the Faculty of Medicine and Health Sciences, Stellenbosch University (Reference numbers: N06/08/148; N13/08/115; S19/05/098). All procedures related to this research complied with the relevant national and institutional committees on human experimentation and with the Helsinki Declaration of 1975, as revised in 2008. Prior to study entry into the parent studies, all participants provided written informed consent and in the case of minors, assent was provided by the minor and consent by the legal guardian or parent. Participants also agreed to future data analysis of their study data.

### Statistical analysis

For this secondary data analysis, statistical analyses were performed on a larger sample (*n* = 111) using the Statistical Package for Social Sciences (SPSS), IBM software (statistics 25). Categorical characteristics were described using cross-tabulation and frequency tables. Normally distributed numerical data were described as the mean along with standard deviation (s.d.) and the median along with the inter-quartile range. Given the skewed distribution of the clinical variable, DUP and the childhood trauma subtype sexual abuse, Spearman’s rank correlation was performed. The linear relationships between childhood trauma exposure and premorbid adjustment scores were assessed using Pearson’s correlation coefficients. Univariate and bivariate analyses were used to identify relevant predictors of distinct social and academic domains of premorbid adjustment, which were entered as independent variables into sequential hierarchical regression models. The domains of premorbid adjustment were the outcome (dependant) variables. We used separate linear regression analyses to assess whether the timing of childhood trauma had a moderating effect on each of the developmental stages of premorbid functioning and childhood trauma (overall and subscale). Statistical significance was set at *p* < 0.05.

## Results

Table 1 provides details of the 111 patients. They were predominantly male, with a diagnosis of schizophrenia or schizophreniform disorder. High levels of all of the abuse subtypes were reported, with 52 (47%) falling into the overall high childhood trauma category and 34 (31%) reporting trauma exposure < 12 years. Table 2 summarises the correlations between childhood trauma exposure and the premorbid adjustment domains.

**TABLE 1:** Socio-demographic and clinical characteristics of the cohort (*n* = 111).

Variables	*n*	%	Mean	s.d.	Median	Range
**Socio-demographic characteristics**						
Age at study entry in years	-	-	24.9	6.9	-	-
Male	77	69.4	-	-	-	-
**Diagnosis**						
Schizophrenia	76	68.5	-	-	-	-
Schizophreniform disorder	34	30.6	-	-	-	-
Schizoaffective disorder	1	0.90	-	-	-	-
Years of education	-	-	10	2.1	-	-
Age of childhood trauma	-	-	9.31	3.85	-	-
Substance use	54	48.6	-	-	-	-
Duration of untreated psychosis in weeks	-	-	41.8	60.8	-	-
Total baseline PANSS score	-	-	93.2	17.2	-	-
Overall high childhood trauma category	52	56	-	-	-	-
Trauma exposure < 12 years	34	64	-	-	-	-
**CTQ**						
Emotional abuse (*n* = 107)	-	-	-	-	11.00	5–24
Physical abuse (*n* = 103)	-	-	-	-	7.00	5–24
Sexual abuse (*n* = 107)	-	-	-	-	5.00	5–25
Emotional neglect (*n* = 101)	-	-	-	-	9.00	5–23
Physical neglect (*n* = 102)	-	-	-	-	9.00	5–22
Total CTQ score (*n* = 96)	-	-	-	-	44.00	25–92
**PAS scores**						
**Childhood (up to 11 years)**						
Sociability and withdrawal (*n* = 111)	-	-	-	-	0.00	0–6
Peer relationships (*n* = 111)	-	-	-	-	1.00	0–6
Scholastic performance (*n* = 111)	-	-	-	-	3.00	0–6
Adaption to school (*n* = 111)	-	-	-	-	0.00	0–6
PAS total childhood (*n* = 111)	-	-	-	-	0.33	0–1.50
**Early adolescence (12–15 years)**						
Sociability and withdrawal (*n* = 111)	-	-	-	-	0.00	0–6
Peer relationships (*n* = 111)	-	-	-	-	2.00	0–6
Scholastic performance (*n* = 108)	-	-	-	-	3.00	0–6
Adaption to school (*n* = 108)	-	-	-	-	0.50	0–5
Socio-sexual aspects of life (*n* = 111)	-	-	-	-	1.00	0–6
PAS total early adolescence (*n* = 111)	-	-	-	-	0.58	0–1.28
**Late adolescence (16–18 years)**						
Sociability and withdrawal (*n* = 110)	-	-	-	-	1.00	0–6
Peer relationships (*n* = 110)	-	-	-	-	2.00	0–5
Scholastic performance (*n* = 96)	-	-	-	-	4.00	0–6
Adaption to school (*n* = 98)	-	-	-	-	2.00	0–6
Socio-sexual aspects of life (*n* = 110)	-	-	-	-	2.00	0–6
PAS total late adolescence (*n* = 110)	-	-	-	-	0.67	0–1.69
PAS overall premorbid adjustment (*n* = 111)	-	-	-	-	0.28	0–0.66

s.d., standard deviation; PANSS, Positive and Negative Syndrome Scale; CTQ, Childhood Trauma Questionnaire; PAS, premorbid adjustment score.

**TABLE 2:** Pearson’s and Spearman’s correlations between childhood trauma and the developmental stages of premorbid adjustment.

Variables	Childhood		Early adolescence		Late adolescence	
	Academic	Social	Academic	Social	Academic	Social
Age at study entry	0.09	−0.08	−0.03	−0.09	−0.22*	−0.25**
Gender	−0.13	−0.06	−0.18	−0.01	−0.13	−0.06
Education in years	−0.39**	0.02	−0.59**	−0.05	−0.57**	−0.17
DUP	0.095	−0.084	0.178	−0.115	0.186	−0.021
PANSS Total	0.18	0.08	0.17	0.17	0.22*	0.19
CTQ-EA	0.15	−0.00	0.18	0.02	0.01	0.13
CTQ-PA	0.21*	0.12	0.13	0.19	0.13	0.25*
CTQ-PN	0.29**	0.21*	0.28**	0.25*	0.18	0.20*
CTQ-EN	0.06	0.08	0.09	−0.00	0.03	0.07
CTQ-SA	0.120	0.080	0.216*	0.265**	0.213*	0.329**
CTQ-Total	0.19	0.09	0.21*	0.13	0.09	0.23*
Age CT	−0.06	−0.08	−0.12	−0.08	−0.13	−0.17

CT, childhood trauma; CTQ, Childhood Trauma Questionnaire; DUP, duration of untreated psychosis; EA, emotional abuse; EN, emotional neglect; PA, physical abuse; PANSS, Positive and Negative Syndrome Scale; PN, physical neglect; SA, sexual abuse.

* , *p* < 0.05;

** , *p* < 0.01.

Analysis of variance (ANOVA) tests were used to assess the relationships between substance use and premorbid adjustment domains.

Academic functioning during both early adolescence (*F* = 4.15, *p* = 0.044) and late adolescence (*F* =7.60, *p* = 0.007) was poorer amongst substance users compared with their non-using counterparts. In the linear regression analyses, we found that the timing of childhood trauma did not significantly moderate the relationship between any stage of premorbid functioning and specific or overall childhood trauma.

### Hierarchical regression to identify predictors of poorer premorbid adjustment

Informed by the statistically significant associations from Pearson’s bivariate correlation analysis, Spearman’s rank correlation analysis and ANOVA test, individual hierarchical linear regression analysis was performed for five of the six outcome measures (PAS childhood academic, PAS early adolescent academic and social, including PAS late adolescent academic and social) (Table 3).

**TABLE 3:** Hierarchical regressions modelling for the effects of childhood trauma as predictors of premorbid adjustment.

Variables	Predictor						
	*R*^2^	*df*	*F*	*p*	β	*t*	*p*
**Overall PAS childhood academic (*n* = 111)**							
Model I	0.14	1	12.63	0.001	-	-	-
Education	-	-	-	-	−0.38	−3.55	0.001
Model II	0.15	3	4.31	0.007	-	-	-
Education	-	-	-	-	−0.35	−3.10	0.003
CTQ-PA	-	-	-	-	0.06	0.50	0.619
CTQ-PN	-	-	-	-	0.04	0.34	0.733
**Overall PAS early adolescence academic (*n* = 108)**							
Model I	0.34	2	18.84	0.000	-	-	-
Education	-	-	-	-	−0.59	−5.75	0.000
Substance use	-	-	-	-	−0.02	−0.16	0.873
Model II	0.35	5	7.26	0.000	-	-	-
Education	-	-	-	-	−0.60	−5.40	0.000
Substance use	-	-	-	-	−0.02	−0.17	0.866
CTQ-PN	-	-	-	-	−0.03	−0.19	0.852
CTQ-SA	-	-	-	-	−0.04	−0.30	0.764
CTQ-total	-	-	-	-	0.03	0.19	0.848
**Overall PAS early adolescence social (*n* = 111)**							
Model I	0.02	1	1.74	0.190	-	-	-
CTQ-SA	-	-	-	-	0.13	1.32	0.190
Model II	0.08	2	4.30	0.016	-	-	-
CTQ-SA	-	-	-	-	0.08	0.84	0.403
CTQ-PN	-	-	-	-	0.26	2.60	0.011
**Overall PAS late adolescence academic (*n* = 110)**							
Model I	0.37	4	10.21	0.000	-	-	-
Age at study entry	-	-	-	-	−0.11	−1.08	0.285
Substance use	-	-	-	-	0.07	0.65	0.515
Education	-	-	-	-	−0.51	−4.92	0.000
PANSS	-	-	-	-	0.11	1.02	0.31
Model II	0.37	5	8.12	0.000	-	-	-
Age at study entry	-	-	-	-	−0.12	−1.12	0.268
Substance use	-	-	-	-	0.07	0.66	0.511
Education	-	-	-	-	−0.51	−4.91	0.000
PANSS	-	-	-	-	0.12	1.09	0.281
CTQ-SA	-	-	-	-	−0.05	−0.49	0.624
**Overall PAS late adolescence social (*n* = 111)**							
Model I	0.05	1	4.82	0.031	-	-	-
Age at study entry	-	-	-	-	−0.23	−2.20	0.031
Model II	0.15	5	3.01	0.015	-	-	-
Age at study entry	-	-	-	-	−0.21	−2.09	0.040
CTQ-PA	-	-	-	-	0.29	1.67	0.099
CTQ-PN	-	-	-	-	0.30	1.87	0.065
CTQ-SA	-	-	-	-	0.16	1.21	0.231
CTQ-Total	-	-	-	-	−0.32	−1.18	0.240

CTQ, Childhood Trauma Questionnaire; PA, physical abuse; PANSS, Positive and Negative Syndrome Scale; PAS, Premorbid Adjustment Scale; PN, physical neglect; SA, sexual abuse, Education, number of completed years of education.

To further investigate the significant associations pertaining to academic functioning in childhood, education was entered into model I. Thereafter, it was adjusted for the childhood trauma variables, physical abuse and neglect in model II. Education demonstrated a significant relationship with childhood academic functioning (*r*^2^ = 0.15, *p* = 0.003). To study the next outcome variable, premorbid academic functioning in early adolescence, education and substance use were entered in model I. Thereafter, it was adjusted for the childhood trauma variables physical neglect and overall childhood trauma in model II. Years of education displayed a significant relationship with academic functioning in early adolescence (*r*^2^ = 0.35, *p* = 0.000). To further study significant associations with social functioning in early adolescence, sexual abuse was entered into model I and adjusted for physical neglect in model II. Physical neglect (*r*^2^ = 0.08, *p* = 0.011) had a significant relationship with premorbid social functioning in early adolescence. To study the variables of interest concerning academic functioning in late adolescence, age at study entry, substance use, years of education and PANNS baseline score were entered into model I, adjusting for the childhood trauma variable, sexual abuse in model II.

Education was the only variable with a significant relevance to academic functioning in late adolescence (*r*^2^ = 0.37, *p* = 0.000). Lastly, to study the outcome variable social functioning in late adolescence, age at study entry was entered into model I, thereafter adjusting for the childhood trauma variables sexual abuse, physical abuse, physical neglect and overall childhood trauma were entered into model II. Age at study entry displayed a significant relationship with social functioning in late adolescence (*r*^2^ = 0.15, *p* = 0.040).

## Discussion

This study examined a cohort of 111 people with FES; physical neglect significantly and independently predicted poorer social premorbid adjustment during early adolescence. Several significant correlations between physical neglect and premorbid functioning across key development periods were also evident. The moderator variable ‘less years of education’ independently predicted poorer premorbid academic functioning during childhood, early adolescence and late adolescence. An earlier age at study entry independently predicted poorer premorbid social functioning in late adolescence. Overall, childhood trauma was not significantly associated with overall premorbid adjustment. In addition, we failed to demonstrate an association between the timing of early life exposure to trauma and premorbid adjustment.

The main finding was that physical neglect significantly and independently predicted poorer social premorbid adjustment during early adolescence. One possible explanation for this is that in a neurodevelopmental disorder such as schizophrenia, neglect in the physical care of the child during periods of cerebral maturation may result in subtle but significant effects on adult social functioning.^42^ The effects of physical neglect on neurodevelopmental pathways have a long-term impact on social performance, coping mechanisms and the stress response.^43^

Furthermore, the beginning of neuro-endocrinological changes characteristic of early adolescence go hand in hand with increased sensitivity to stress. Additional stress related to physical neglect during this period may be implicated in abnormal behavioural development,^44^ with an increased risk of avoidant peer interactions and social withdrawal.^45^ Exposure to physical neglect may therefore contribute to a higher incidence of severe emotional disturbances,^45^ lack of social skills^46^ and unpopularity amongst peers, collectively increasing the risk for social isolation.^45^

No association between the timing of early life exposure to trauma and premorbid adjustment was found in our study. Only one prior study to date has investigated the relationship between the timing of trauma exposure and premorbid adjustment in schizophrenia and found that global functional impairment was greater in those exposed to SPA before the age of 11 than those exposed to SPA at a later age.^11^

One possible reason for the negative finding in our study for an association between the timing of childhood trauma and premorbid functioning is that only a subset of the patients (*n* = 53) provided information on the timing of childhood trauma. A total of 34 patients (64%) reported trauma exposure < 12 years of age and 19 (36%) reported trauma ≥ 12 years of age. This sample may therefore be underpowered to examine the timing of childhood trauma, which could have led to a false-negative finding. Another possible reason for the negative finding is that poverty and neglect are inter-related^47^ and, in a setting where poverty is endemic, timing may be difficult to assess for physical neglect, the childhood trauma subtype significantly associated with premorbid functioning in our study.

We also identified significant correlations between clinical and demographic variables and premorbid functioning across key development periods. Consistent with the findings of Larsen et al.,^23^ lower total years of education predicted poorer premorbid academic functioning in childhood and early adolescence. Education may be a protective factor in schizophrenia, with higher levels of education and complex occupations associated with better outcomes and less cognitive impairment.^20^ In addition, we found that earlier age of disease onset was predictive of poorer social functioning in late adolescence. An earlier age of schizophrenia onset is frequently associated with poorer premorbid social functioning, in particular with social withdrawal and poor peer relationships.^48^

There are some limitations of this study. Inferences on causality cannot be made because of the cross-sectional design of the study. A larger representative sample would provide more power to study the effects of timing of trauma exposure on the relationship between premorbid functioning and childhood trauma and to detect smaller effects. The study did not control for resilience and family support, nor was a healthy control group included. The CTQ-SF is a retrospective instrument that potentially imposes a risk for recall bias and unreliability.

However, Fergusson et al.^49^ established that the retrospective errors of measurement on childhood trauma data do not significantly affect the validity of a study. It was also found that childhood trauma is more frequently reported in severe mental disorders^37^ and therefore we decided to omit the minimisation/denial score in the CTQ-SF. The physical neglect subscale displays poor factor loadings and homogeneity and supplementary assessment measures are recommended.^14^ We included the LEC-5 and Life Events Timeline to verify data on childhood trauma exposure and the timing of events.

The strengths of the study include the incorporation of specific effects of trauma subtypes on the various domains of premorbid adjustment during different developmental periods. This study contributes to the small body of research on the subject matter in FES, and also contributes to the timing of childhood trauma. The use of a hierarchical regression analysis allowed the researcher to control for multiple confounding factors. Lastly, the study emphasised the deleterious consequences of childhood trauma on mental health in a developing country.

## Conclusion

In summary, our study suggests a significant link between physical neglect and premorbid social functioning in early adolescence, which is an important sensitive period of neurodevelopment. Future recommendations would be to conduct larger, representative, longitudinal studies to elucidate the mechanisms involved in the effect of childhood trauma on premorbid adjustment, including long-term outcomes. Inclusion of matched healthy controls when studying childhood trauma histories in relationship with premorbid social and academic functioning could inform on illness-specific effects on premorbid functioning.
